# Determination of Bosentan in Pharmaceutical Preparations by Linear Sweep, Square Wave and Differential Pulse Voltammetry Methods 

**Published:** 2015

**Authors:** Alptug Atila, Bilal Yilmaz

**Affiliations:** *Department of Analytical Chemistry, Faculty of Pharmacy, Ataturk University, 25240, Erzurum, Turkey.*

**Keywords:** Bosentan, Cyclic voltammetry, Linear sweep voltammetry, Square wave voltammetry, Differential pulse voltammetry

## Abstract

In this study, simple, fast and reliable cyclic voltammetry (CV), linear sweep voltammetry (LSV), square wave voltammetry (SWV) and differential pulse voltammetry (DPV) methods were developed and validated for determination of bosentan in pharmaceutical preparations. The proposed methods were based on electrochemical oxidation of bosentan at platinum electrode in acetonitrile solution containing 0.1 M TBACIO_4_. The well-defined oxidation peak was observed at 1.21 V. The calibration curves were linear for bosentan at the concentration range of 5-40 µg/mL for LSV and 5-35 µg/mL for SWV and DPV methods, respectively. Intra- and inter-day precision values for bosentan were less than 4.92, and accuracy (relative error) was better than 6.29%. The mean recovery of bosentan was 100.7% for pharmaceutical preparations. No interference was found from two tablet excipients at the selected assay conditions. Developed methods in this study are accurate, precise and can be easily applied to Tracleer and Diamond tablets as pharmaceutical preparation.

## Introduction

**Figure 1:**
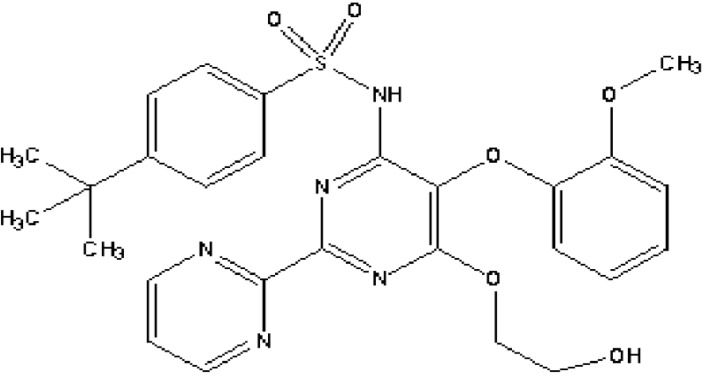
Chemical structure of bosentan

Bosentan, (4-*tert*-butyl-*N*-[6-(2-hydroxyethoxy)-5-(2-methoxyphenoxy)-2-(pyrimidin-2-yl) pyrimidin-4-yl], is a competitive oral dual endothelin receptor antagonist which is non-selective for endothelin A and B receptors (Figure 1). Bosentan is being used as an oral drug for the treatment of pulmonary arterial hypertension (1, 2). It has a high protein binding rate (98%), especially to albumin and is rapidly absorbed after oral administration. Its bioavailability is 45-50%. The peak plasma concentration occurs within 3-5 h (3, 4). Bosentan is eliminated mainly by hepatic metabolism which renal elimination occurs for only 0.9% of the administered dose (5).

It is the first orally active drug approved by United States Food and Drug Administrative as Tracleer (125 mg) for the successful treatment of pulmonary arterial hypertension. Tracleer improves the exercise ability and decreases the rate of clinical worsening in patients with WHO Class III or IV symptoms of pulmonary arterial hypertension, by blocking the binding of endothelin to its receptors, thereby negating endothelin’s deleterious effects (6-8). Further Tracleer has been demonstrated to be effective in remodeling the pulmonary vascular tree through several mechanisms including vasodilatation, antifibrotic and antithrombotic actions (9).

An extensive literature survey revealed that there are several HPLC methods for the determination of bosentan monohydrate and its metabolite in blood plasma, whereas, there are few other literatures disclosed only for the quantitative determination of bosentan in pharmaceutical formulation samples (10-13). 

The reported methods were influenced by interference of endogenous substances and potential loss of drugs in the re-extraction procedure and involving lengthy, tedious and time-consuming plasma sample preparation and extraction processes and requiring a sophisticated and expensive instrumentation. 

Bosentan is a new drug. There is not official in any pharmacopoeia. The development of a new method capable of determining drug amount in pharmaceutical dosage forms is important. Electroanalytical techniques have been used for the determination of a wide range of drug compounds with the advantages that there are, in most, instances no need for derivatization and that these techniques are less sensitive to matrix effects than other analytical techniques. Additionally, application of electrochemistry includes the determination of electrode mechanism. Redox properties of drugs can give insights into their metabolic fate or their *in-vivo* redox processes or pharmacological activity (14-17). Despite the analytical importance of the electrochemical behavior and oxidation mechanism of bosentan, no report has been published on the voltammetric study of the electrochemical oxidation of bosentan in nonaqueous media. It is well known that the experimental and instrumental parameters directly affect the electrochemical process and voltammetric response of drugs. Consequently, it would be interest to investigate the oxidation process of bosentan in aprotic media. Therefore, the goal of this work was the development of new LSV, SWV and DPV methods for the direct determination of bosentan in pharmaceutical preparations without any time-consuming extraction or evaporation steps prior to drug assay. This paper describes fully validated simple, rapid, selective and sensitive procedures for the determination of bosentan employing LSV, SWV and DPV methods the platinum disc electrode. Besides, the methods were successfully applied for the quality control of two commercial bosentan tablets form to quantify the drug and to check the formulation content uniformity.

## Experimental

*Chemical* *and reagents*

Bosentan was obtained from Actelion Pharmaceuticals (Allschwil, Switzerland). Acetonitrile (Fluka for HPLC analysis) was purified by drying with calcium hyride, followed by distillation from phosphorus pentoxide. After purification in order to eliminate its water content as much as possible, it was kept over molecular sieves (3Å, Merck). Tetrabutylammonium perchlorate (TBAClO_4_) were purchased from Fluka and used as received without further purification. Tracleer and Diamond tablets (125 mg bosentan) were obtained from pharmacy (Erzurum, Turkey).

*Electrochemical instrumentation*

Electrochemical experiments were performed on a Gamry Potentiostat Interface 1000 controlled with software PHE 200 and PV 220. All measurements were carried out in a single-compartment electrochemical cell with a standard three-electrode arrangement. A platinum disk with an area of 0.72 cm^2^ and a platinum wire were used as the working and the counter electrodes, respectively. The working electrode was successively polished with 1.0, 0.3 and 0.05 µm alumina slurries (Buehler) on microcloth pads (Buehler). After each polishing, the electrode was washed with water and sonicated for 10 min in acetonitrile. Then, it was immersed into a hot piranha solution (3:1, H_2_SO_4_, 30% H_2_O_2_) for 10 min, and rinsed copiously with water. Caution: Piranha is a vigorous oxidant and should be used with extreme caution! All potentials were reported versus Ag/AgCl/KCl (3.0 M) reference electrode (BAS Model MF-2078) at room temperature. The electrolyte solutions were degassed with purified nitrogen for 10 min before each experiment and bubbled with nitrogen during the experiment. Operating conditions for SWV were pulse amplitude 25 mV, frequency 15 Hz, potential step 4 mV; and for DPV were pulse amplitude 50 mV, pulse width 50 ms, scan rate 20 mV/ s.

*Preparation of the standard and quality control solutions *

The stock standard solution of bosentan was prepared in 0.1 M TBAClO_4_/acetonitrile μ C. Working standard solutions were prepared from the stock solution. Standard solutions were prepared as 5-40 µg/mL for LSV and 5-35 µg/mL for SWV and DPV, respectively. The quality control (QC) solutions were prepared by adding aliquots of standard working solution of bosentan to final concentrations of 7.5, 17.5 and 37.5 μg /mL for LSV and 7.5, 17.5 and 32.5 μg/mL for SWV and DPV. 

*Procedure for pharmaceutical preparations*

A total 10 tablets of bosentan (Tracleer and Diamond) accurately weighed and powdered. An amount of this powder corresponding to one tablet bosentan content was weighed and accurately transferred into 100 mL calibrated flask and 50 mL of 0.1 M TBAClO_4_/acetonitrile was added and then the flask was sonicated to 10 min at room tempature. The flask was filled to volume with 0.1 M TBAClO_4_/acetonitrile. The resulting solutions in both the cases were filtered through Whatman filter paper no 42 and suitably diluted to get final concentration within the limits of linearity for the respective proposed methods. The drug content of bosentan tablets were calculated from the current potential curves. 

## Results and Discussion

*Voltammetric behavior of* *bosentan*

**Figure 2:**
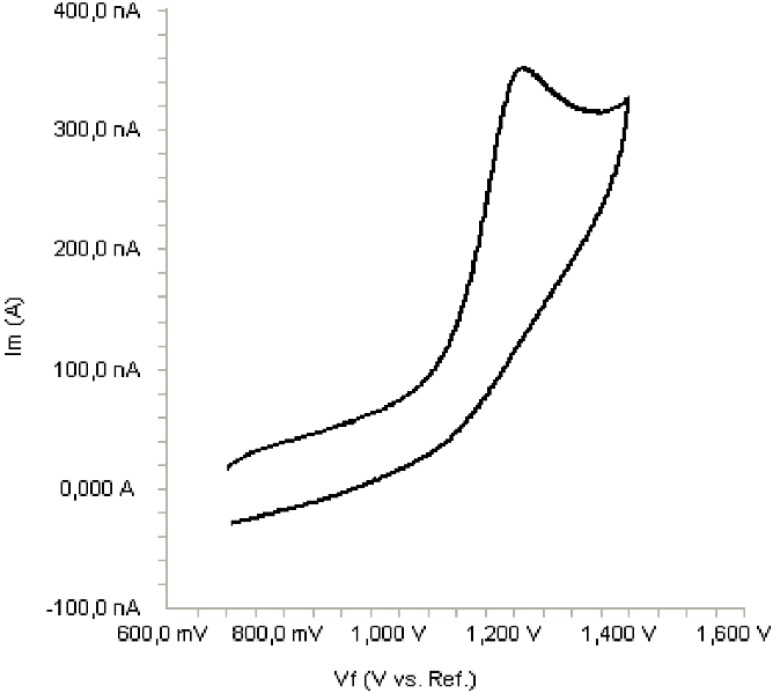
Cyclic voltammogram for the oxidation of 20 μg/mL bosentan in acetonitrile containing 0.1 M TBAClO_4_ at Pt disk electrode, scan rate: 0.1 V s^-^^1^.

The electrochemical behavior of bosentan was investigated at the Pt disc electrode in anhydrous acetonitrile solution containing 0.1 M TBAClO_4 _as the supporting electrolyte by using cyclic voltammetry (CV). Figure 2 shows a typical cyclic voltammogram of 20 μg/mL bosentan recorded under these conditions for the scan rate of 0.1 V/s. In the anodic sweep, an oxidation peak is seen at about potential of 1.21 V. Upon reversing the potential scan, no reduction peak corresponding to this oxidation wave is observed, indicating the irreversible nature of the electrode reactions. 

**Figure 3:**
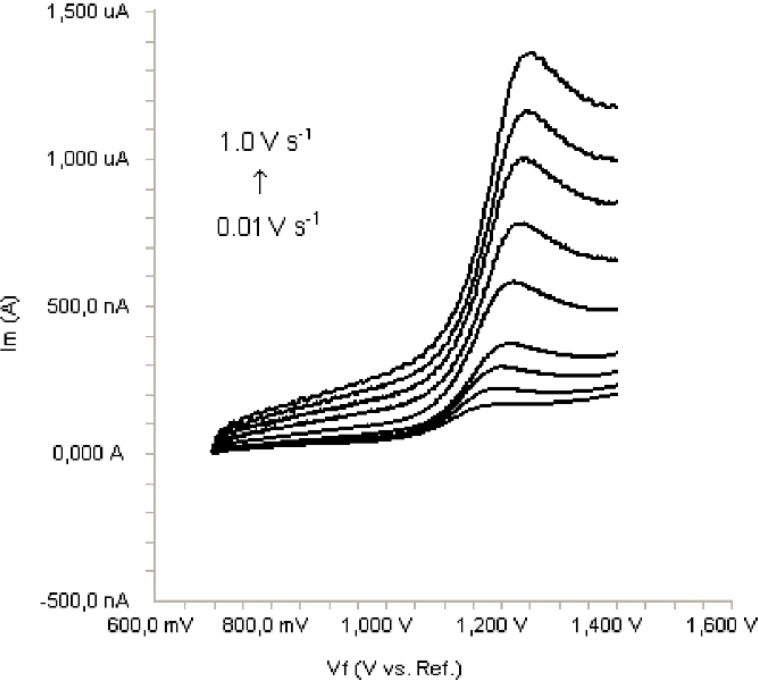
Linear sweep voltammograms for the oxidation of 20 μg/mL bosentan in acetonitrile containing 0.1 M TBAClO_4 _as a function of scan rate.

**Figure 4:**
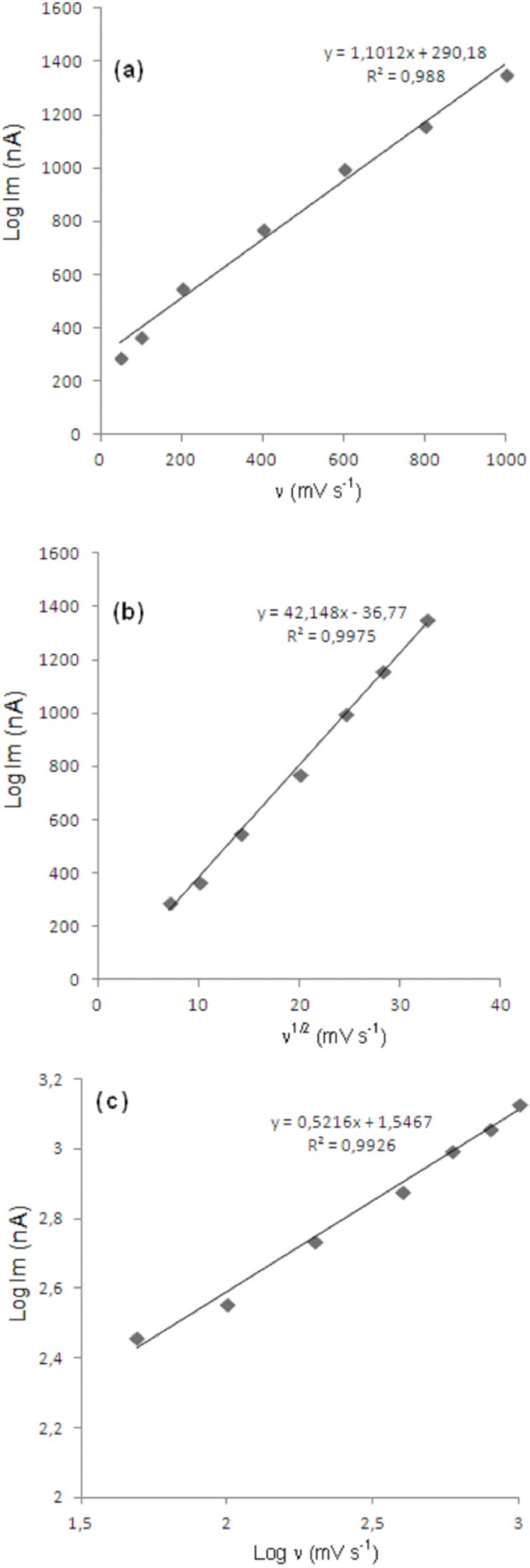
Dependence of peak current on the scan rate (20 μg/mL).

In order to gain a deeper insight into the voltammetric waves, the effect of scan rate on the anodic peak currents (*İ*_m_) and peak potentials (E_p_) was studied in the range of 0.01-1 V/s of the potential scan rates in acetonitrile solution containing 20 μg/mL concentration of bosentan (Figure 3). The representative linear sweep voltammograms obtained at Pt electrode for 20 μg/mL bosentan as a function of the scan rate are presented in Figure 4. Scan rate dependency experiments show that the peak currents for peak vary linearly with the scan rate (ν) (Figure 4a,b), which points out the adsorption-controlled process. However, the plots of logarithm of peak currents versus logarithm of scan rates for 20 μg/mL concentration of bosentan display straight lines with 0.52 slope (Figure 4c), which are close to theoretical value of 0.5 expected for an ideal diffusion-controlled electrode process (18). Log I_m_-log ν curve is more eligible for this aim, therefore, a diffusional process for peak should be considered. These results suggest that the redox species are diffusing freely from solution and not precipitating onto the electrode surface. The reason for this behavior may be due to the solubility of the intermediate species in acetonitrile or poor adherence of products on the electrode surface.

**Figure 5:**
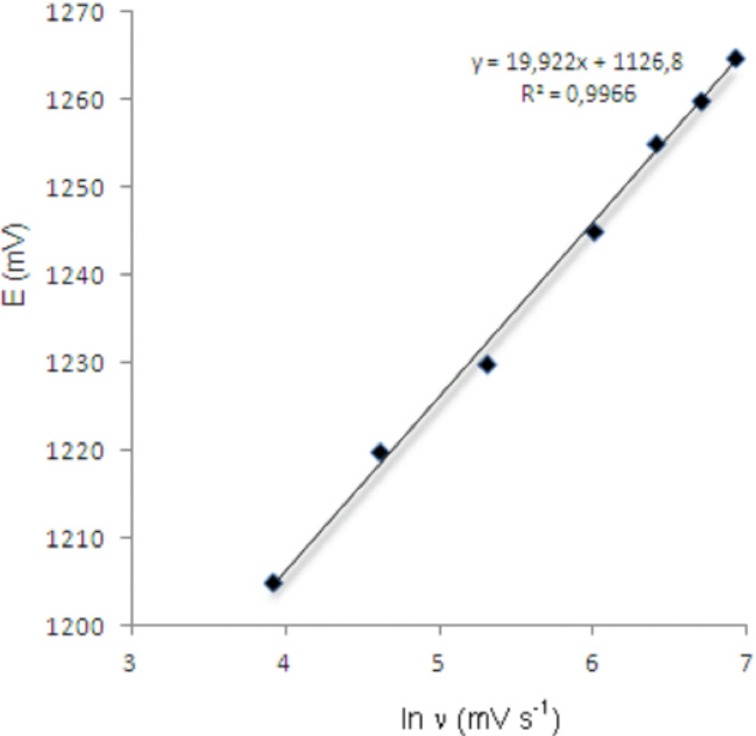
Dependence of anodic peak potentials of voltammetric peak for the oxidation of 20 μg/mL bosentan on the scan rate.

As shown in Figure 3, the oxidation peak potential (E_pa_) for peaks shift toward more positive values with increasing scan rate. The relationship between the peak potential and scan rate is described by the following equation (19), Epa=Eδ+RT/[1-αnaF][0.78+lnD12Ks-1-0.5lnRT/[(1-α)naF]]+RT/[(1-α)naF]/2ln⁡ v and from the variation of peak potential with scan rate αn_a_ can be determined, where α is the transfer coefficient and n_a _is the number of electrons transferred in the rate determining step. According to this equation, the plots of the peak potentials versus ln ν for oxidation peak show linear relationship (Figure 5). The slope indicate the value of αn_a_ is 0.64 for peak. Also, this value obtained indicate the total irreversibility of the electron transfer processes. This result show that the chemical step is a fast following reaction coupled to a charge transfer.

*Validation of the method*

The validation was carried out by establishing specificity, linearity, accuracy, precision, limit of detection (LOD), limit of quantification (LOQ), recovery, ruggedness according to ICH Q2B recommendations (19, 20).

*Specificity*

The effects of common excipients and additives were tested for their possible interferences in the assay of bosentan. The simulated and placebo samples were prepared and analyzed. It has not been determined any interference of these substances at the levels found in dosage forms. Excipient that was used in this preparation was the most commonly used by the pharmaceutical industry. The presence of titanium dioxide, talc, lactose, starch, and magnesium stearate did not appear interfere in the results of the analysis.

*Linearity*

**Figure 6:**
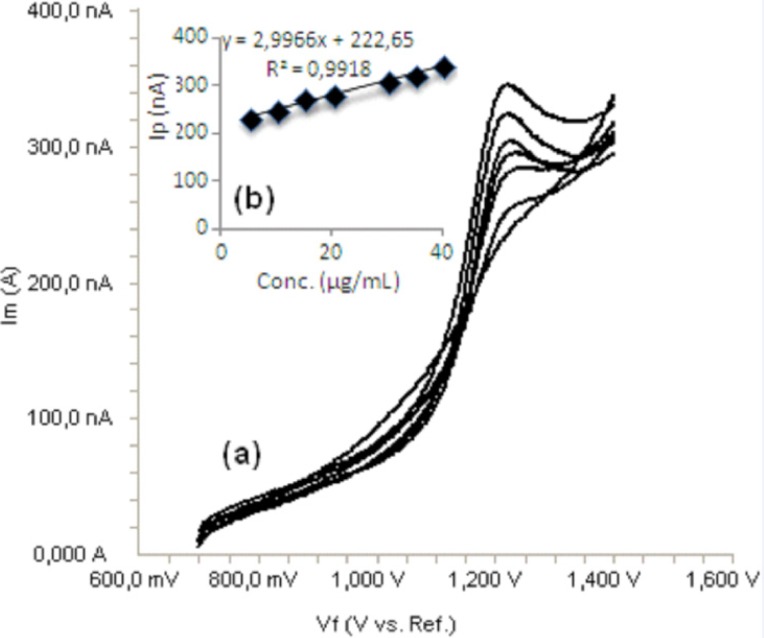
a) Linear sweep voltammograms for different concentrations of bosentan in acetonitrile solution containing 0.1 M TBACIO_4_ (5, 10, 15, 20, 30, 35 and 40 μg/mL), b) Mean calibration graph (n=6)

**Figure 7:**
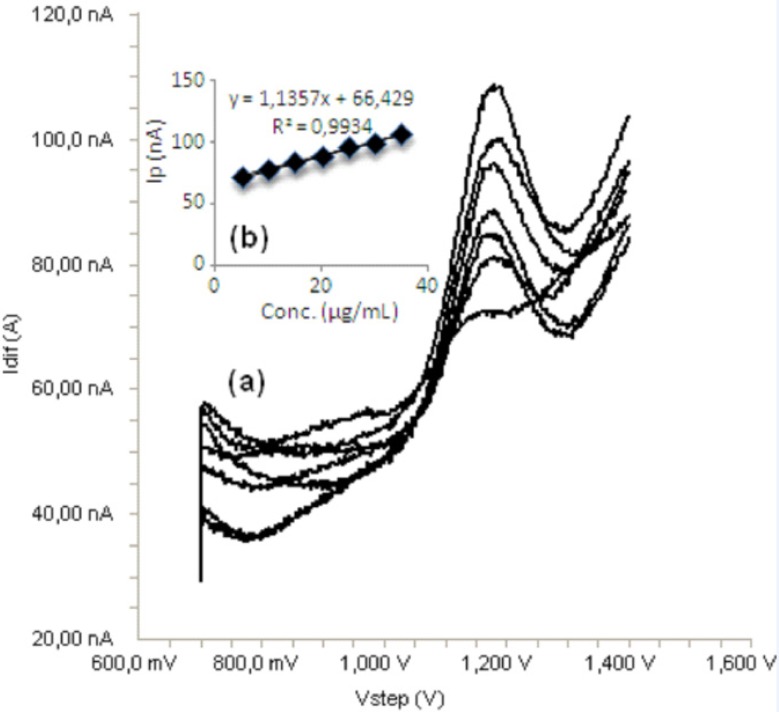
a) Square wave voltammograms for different concentrations of bosentan in acetonitrile solution containing 0.1 M TBACIO_4_ (5, 10, 15, 20, 25, 30 and 35 μg/mL), b) Mean calibration graph (n=6)

**Figure 8:**
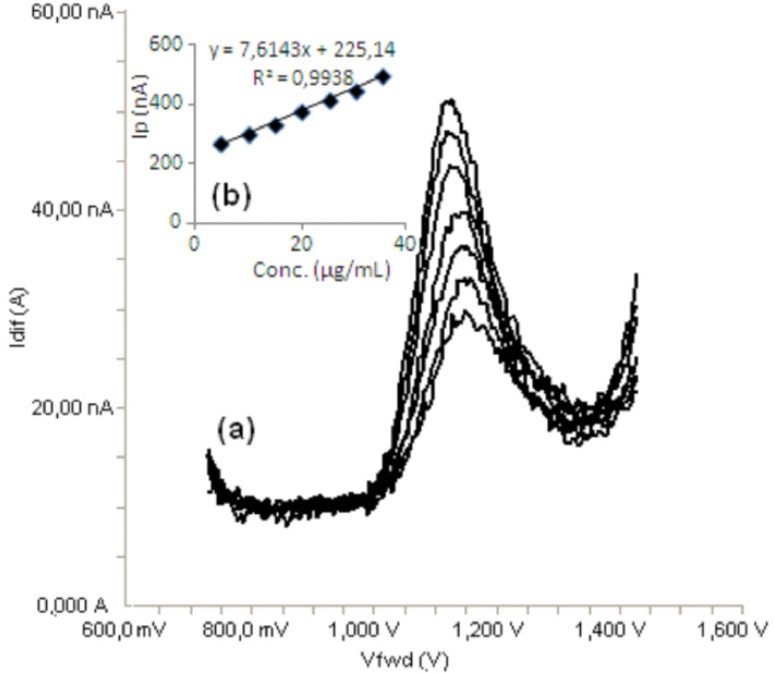
a) Differential pulse voltammograms for different concentrations of bosentan in acetonitrile solution containing 0.1 M TBACIO_4_ (5, 10, 15, 20, 25, 30 and 35 μg/mL), b) Mean calibration graph (n=6)

Standard solutions were prepared as 5-40 μg/mL (5, 10, 15, 20, 30, 35 and 40) for LSV (Figure 6) and 5-35 μg/mL (5, 10, 15, 20, 25, 30 and 35) for SWV and DPV (Figures 7, 8), respectively. Calibration curves were constructed for bosentan standard by plotting the concentration of compound versus peak current responses. The calibration curves were evaluated by its correlation coefficients. The correlation coefficients *(r)* of all the calibration curves were consistently greater than 0.99. The linear regression equations were calculated by the least squares method using Microsoft Excel^®^ program and summarized in Table 1.

**Table 1:** Linearity of bosentan

**Method**	**Range** **(µg/mL)**	**LR**	**Sa**	**S**_b_	**R** ^2^	** LOD** **(µg/mL) **	** LOQ** **(µg/mL) **
LSV	5-40	y=2.9966x+222.65	1.45	0.057	0.9918	1.6	4.8
SWV	5-35	y=1.1357x+66.429	0.31	0.023	0.9934	0.9	2.7
DPV	5-35	y=7.6143x+225.14	0.69	0.068	0.9938	0.3	0.9

^ a ^Based on three calibration curves, LR: linear regression, S_a_: standard deviation of intercept of regression line, S_b_: Standard deviation of slope of regression line, R^2^: Coefficient of correlation, y: peak current, x: bosentan concentration, LOD: Limit of detection, LOQ: limit of quantification

*Accuracy and precision*

Accuracy of the assay methods were determined for both intra-day and inter-day variations using the six times analysis of the quality control (QC) samples. Precision of the assay was determined by repeatability (intra-day) and intermediate precision (interday). Repeatability refers to the use of the analytical procedure within a laboratory over a short period of time that was evaluated by assaying the QC samples during the same day. Intermediate precision was assessed by comparing the assays on different days (3 days). The intra-day accuracy ranged from 2.26% to 6.29% and precision from 1.72% to 5.34% (Table 2). The results obtained from intermediate precision (inter-day) also indicated a good method precision. All the values were within the acceptance criteria of 6.29%. 

**Table 2:** Precision and accuracy of bosentan

		** Intra-day**			**Inter-day**		
**Method**	**Added** **(µg/mL)**	**Found±SD** a	**Precision** **% RSD** b	**Accuracy** c	**Found±SD** a	**Precision** **% RSD** b	**Accuracy** c
	7.5	7.29±0.19	2.61	-2.80	7.88± 0.23	2.91	5.06
LSV	17.5	18.60±0.32	1.72	6.29	18.13±0.30	1.65	3.60
	37.5	35.50±0.79	2.22	-5.33	39.44±0.81	2.05	5.17
	7.5	7.67±0.41	5.34	2.26	7.31±0.31	4.24	-2.53
SWV	17.5	17.95±0.62	3.45	2.57	16.47±0.81	4.92	-5.88
	32.5	31.40±1.39	4.42	-3.38	33.20±1.29	3.89	2.15
	7.5	7.69±0.25	3.25	2.53	7.79±0.34	4.36	3.86
DPV	17.5	16.81±0.71	4.22	-3.94	18.42±0.77	4.18	5.25
	32.5	34.18±0.89	2.60	5.17	33.69±0.65	1.93	3.66

a SD: Standard deviation of six replicate determinations,

b RSD: relative standard deviation, Average of six replicate determinations

c Accuracy: (%relative error) (found-added)/addedx100

*Limits of Detection (LOD) and Quantification (LOQ)*

The LOD and LOQ of bosentan by the proposed methods were determined using calibration standards. LOD and LOQ values were calculated as 3.3 *σ*/*S *and 10 *σ*/*S*, respectively, where *S *is the slope of the calibration curve and *σ *is the standard deviation of *y*-intercept of regression equation (*n=*6) (21). The LOD and LOQ values of the methods were summarized in Table 1.

*Recovery*

To determine the accuracy of the LSV, SWV and DPV methods and to study the interference of formulation additives, the recovery was checked as three different concentration levels. Analytical recovery experiments were performed by adding known amount of pure drugs to pre-analyzed samples of commercial tablet forms. The recovery values were calculated by comparing concentration obtained from the spiked samples with actual added concentrations. These values are also listed in Table 3. 

**Table 3:** Recovery of bosentan in pharmaceutical preparations

**Commercial preparation**	**Method**	**n**	**Found (mg) Mean±SD**	**Recovery**	**% RSD** a	**Confidence ınterval**
Tracleer (125 mg/tablet)	LSV	6	125.9±2.10	100.7	1.66	123.5- 126.8
	SWV	6	127.3±4.44	101.8	3.48	122.1-129.4
	DPV	6	124.5±1.39	99.6	1.12	121.9-126.7
Diamond (125 mg/tablet)	LSV	6	126.2±3.21	101.0	2.54	124.6-127.4
	SWV	6	127.9±2.53	102.3	1.97	123.9-128.1
	DPV	6	125.7±2.22	100.6	1.76	123.1-127.9

SD: Standard deviation of six replicate determinations, RSD: relative standard deviation,

a Average of six replicate determinations

*Ruggedness*

In this study, the LSV, SWV and DPV determination of bosentan were carried out by a different analyst in same instrument with the same standard (Table 4). The results showed no statistical differences between different operators suggesting that the developed method was rugged.

**Table 4:** The results of analyses of bosentan by a different analyst^a^.

**Method**	**Added** **(µg/mL)**	**Found (µg/mL) Mean±SD**	**% Recovery**	**% RSD** a
	5	4.9 ± 0.13	98.0	2.65
LSV	15	14.8 ± 0.27	98.7	1.82
	35	35.4 ± 0.73	101.1	2.06
	5	5.1 ± 0.18	102.0	3.53
SWV	15	14.8 ± 0.25	98.7	1.69
	35	35.2 ± 1.67	100.6	4.74
	5	5.2 ± 0.21	104.0	4.04
DPV	15	14.6 ± 0.28	97.3	1.92
	35	35.6 ± 1.02	101.7	2.87

a Mean measurements of six replicate determinations

*Stability*

To evaluate the stability of bosentan, standard solutions were prepared separately at concentrations covering the low, medium, and higher ranges of calibration curves for different temperature and times. These solutions were stored at room temperature, refrigerated (4 ^0^C) and frozen (-20 ^0^C) temperature for 24 h and 72 h. The stability of bosentan was obtained within the acceptance range of 90-110%.

*Comparison of the methods*

LSV, SWV and DPV voltammetry methods were applied for the determination of the commercial tablets (Table 3). The results show that high reliability and reproducibility of three methods. The best results were statistically compared using the F-test. At 95% confidence level, the calculated F-values do not exceed the theoretical values (Table 5). Therefore, there are no significant difference between LSV, SWV and DPV voltammetry methods. 

The proposed methods were compared with HPLC method (22) in literature. In this study, the concentration of bosentan was determined on a Waters 2695 HPLC system on a reverse phase Agilent XDB C18 column (150 mm × 4.6 mm, *i.d*., 5 μm) using a mobile phase mixture containing phosphate buffer (pH 5) and acetonitrile in 45:55% v/v ratio. The flow rate was 1.0 mL/min and column effluents were monitored at 270 nm and bosentan eluted at 5.7 minutes. The method is linear in the concentration range of 25-150 μg/mL. In this present work, developed LSV, SWV and DPV methods have small linearity range (5-40 μg/mL for LSV and 5-35 μg/mL for SWV and DPV). As the LOQ of the proposed the methods are lower than the earlier reported works (22,23). 

Besides, the results of the proposed methods were statistically compared with those obtained by the reported method (22). Statistical analysis of the results revealed no significant difference between the performance of the proposed and reference method using variance ratio F test (Table 5). The results obtained showed that the calculated F-values did not exceed the theoretical values from which we can conclude that the proposed method do not differ significantly from HPLC method.

**Table 5:** Comparison of the proposed and reported methods for determination of bosentan

**Parameters**	**LSV**	**SWV**	**DPV**	**Reported** ** method [22]**
Mean (recovery%)	100.80	101.85	100.85	99.97
SD	2.66	2.49	1.81	0.1051
%RSD	2.64	2.44	1.79	0.1198
Variance	7.08	5.95	3.20	0.0110
F-test	4.02	3.78	3.18	**-**

SD: Standard deviation of six replicate determinations, RSD: relative standard deviation, Theoretical values at p=0.05

Ho hypothesis: no statistically significant difference exists between four methods

*F*_t_ >F_c_: Ho hypothesis is accepted (P > 0.05)

## Conclusion

In this study, the electrochemical behavior of bosentan has been studied in nonaqueous media by CV, LSV, SWV and DPV voltammetry methods. It has concluded that there is a completely diffusion-controlled current process which isn't affected by adsorption phenomenon. Besides, in the present report, a simple, rapid, sensitive, reliable, specific, accurate and precise LSV, SWV and DPV methods for the determination of bosentan in pharmaceutical preparations were developed and validated. The method described has been effectively and efficiently used to analyze bosentan pharmaceutical tablets without any interference from the pharmaceutical excipients. The voltammetric run time of 5 min allows the analysis of a large number of samples in a short period of time. Therefore, the methods can be used effectively without separation for routine analysis of bosentan in pure form and its formulations.
